# Point-of-care neutrophil CD64 as a rule in diagnostic test for bacterial infections in the emergency department

**DOI:** 10.1186/s12873-023-00800-2

**Published:** 2023-03-14

**Authors:** N. L. M. van de Ven, S. H. Bongers, R. Spijkerman, L. Koenderman, L. P. H. Leenen, F. Hietbrink, The COVPACH study group, Thomas M. P. Nijdam, Bas J. J. Bindels, Nikita K. N. Jorritsma, Remi Verhaegh, Judith S. Spanjaard, Benjamin W. Verboeket, Duco Laane, Karlijn van Wessem, Wiebe Buitenwerf, Daan E. J. van Spengler, Eva Mulder, Nienke Vrisekoop, Harry Heijerma, Harriët M. R. van Goor, Amely Daza Zabaleta, Frederiek van den Bos, Feikje Stiphout, Karin A. H. Kaasjager, Emma Rademaker, Meri R. J. Varkila, Nikki de Mul, Olaf L. Cremer, Arjen Slooter, Maarten Limper, Helen Leavis, Eveline M. Delemarre, Aridaman Pandit, Femke van Wijk, Stefan Nierkens, Bernard N. Jukema, Chantal C. Clark, Arjan D. Barendrecht, Cor W. Seinen, Sandra Drost-Verhoef, Simone Smits, Naomi M. J. Parr, Sylvie A. E. Sebastian, Arnold C. Koekman, Annet C. van Wesel, Erhard van der Vries, Coen Maas, Steven de Maat, Saskia Haitjema, Imo E. Hoefer, Gerjen H. Tinnevelt, Jeroen J. Jansen

**Affiliations:** 1 Department of Trauma Surgery, University Medical Center Utrecht, Utrecht, The Netherlands; 2 Center for Translational Immunology, University Medical Center Utrecht, Utrecht, The Netherlands; 3 Department of Respiratory Medicine, University Medical Center Utrecht, Utrecht, The Netherlands

**Keywords:** nCD64, Bacterial infections, Viral infections, COVID-19, Inflammation, Point of care immunology

## Abstract

**Introduction:**

Bacterial infections are frequently seen in the emergency department (ED), but can be difficult to distinguish from viral infections and some non-infectious diseases. Common biomarkers such as c-reactive protein (CRP) and white blood cell (WBC) counts fail to aid in the differential diagnosis. Neutrophil CD64 (nCD64), an IgG receptor, is suggested to be more specific for bacterial infections. This study investigated if nCD64 can distinguish bacterial infections from other infectious and non-infectious diseases in the ED.

**Methods:**

All COVID-19 suspected patients who visited the ED and for which a definitive diagnosis was made, were included. Blood was analyzed using an automated flow cytometer within 2 h after presentation. Patients were divided into a bacterial, viral, and non-infectious disease group. We determined the diagnostic value of nCD64 and compared this to those of CRP and WBC counts.

**Results:**

Of the 291 patients presented at the ED, 182 patients were included with a definitive diagnosis (bacterial infection *n* = 78; viral infection *n* = 64; non-infectious disease *n* = 40). ROC-curves were plotted, with AUCs of 0.71 [*95%CI:* 0.64–0.79], 0.77 [0.69–0.84] and 0.64 [0.55–0.73] for nCD64, WBC counts and CRP, respectively. In the bacterial group, nCD64 MFI was significantly higher compared to the other groups (*p* < 0.01). A cut-off of 9.4 AU MFI for nCD64 corresponded with a positive predictive value of 1.00 (sensitivity of 0.27, a specificity of 1.00, and an NPV of 0.64). Furthermore, a diagnostic algorithm was constructed which can serve as an example of what a future biomarker prediction model could look like.

**Conclusion:**

For patients in the ED presenting with a suspected infection, nCD64 measured with automatic flow cytometry, has a high specificity and positive predictive value for diagnosing a bacterial infection. However, a low nCD64 cannot rule out a bacterial infection. For future purposes, nCD64 should be combined with additional tests to form an algorithm that adequately diagnoses infectious diseases.

**Supplementary Information:**

The online version contains supplementary material available at 10.1186/s12873-023-00800-2.

## Introduction

Inflammatory diseases, including infections, are commonly diagnosed in the Emergency Department (ED). Several established markers aid the physician to reduce the differential diagnoses. CRP and WBC counts are often measured in cases suspected of a bacterial or viral infection. However, these markers are also elevated in patients due to various other inflammatory reasons, such as trauma or recent surgery [1]. These biomarkers individually have been shown to be unable to differentiate between viral and bacterial infections [2–4]. Consequently, CRP and WBC counts are insufficient at either ruling in or ruling out bacterial infections in comparison to other inflammatory diseases.

Flow cytometry was previously unavailable as a diagnostic tool in the ED, because of several limitations. Conventional flow cytometry is labor-intensive and innate immune cells like neutrophils are known to quickly become activated as a result of ex vivo manipulation, which hampers reliability [5, 6]. Both of these limitations are overcome by automated flow cytometry [5]. A customized fully automated flow cytometer recently became available in the ED [5]. The AQUIOS CL® allows fast (within 15–30 minutes) and reliable automated Point-of-Care (PoC) analysis and the results are highly reproducible, as ex vivo manipulation is minimized [5].

CD64 is a high-affinity IgG receptor, also known as FcɣR1. In homeostatic situations, CD64 is present on macrophages and monocytes, and only in a low amount on neutrophils [7]. In vivo, neutrophil CD64 (nCD64) is upregulated within 1–6 hours after administration of pro-inflammatory cytokines such as IFN-gamma and G-CSF or bacterial cell wall components such as LPS [8, 9]. In the absence of these pro-inflammatory factors, nCD64 levels start to decrease after 48 hours and are assumed to return to baseline within 7 days [9]. nCD64 is also upregulated in viral infections, but to a much lower degree [10]. As such, nCD64 is seen as a potential biomarker for diagnosing bacterial infections [7, 11]. Some previous research studied the diagnostic efficiency of nCD64 in local infections [12]. Most research focused on more severe bacterial infections, such as sepsis, and mainly in patients admitted to an intensive care unit (ICU) [13–15]. Several meta-analyses suggest a potential added value of nCD64 for early diagnosis of sepsis in the ICU. Repeated measurements over several days might even improve the diagnostic yield of such a test [15]. However, these patients were all severely ill and already in the ICU and therefore the situation is not fully comparable to the situation and diagnostic dilemma in the ED.

In the ED, fast differentiation between non-infectious disease and viral or bacterial (super) infection is of paramount importance, especially during a pandemic. Therefore, we analyzed the diagnostic yield of automated nCD64 analysis in all patients with suspected infections presenting at the ED during the first wave of COVID-19 in The Netherlands.

## Methods

### Study design

This study was part of a single-center prospective observational study conducted in the University Medical Center Utrecht (UMCU, Utrecht, the Netherlands) during the first wave of the COVID-19 pandemic, as described elsewhere in detail by Spijkerman et al. [16] For this study a waiver for formal ethical approval was provided by the Medical Research Ethics Committee (MREC) Utrecht under protocol number 20–284/C. Therefore, a Biobank procedure was initiated and approved (protocol nr. 20–175), for which an opt-out procedure was in place. A waiver for informed consent to participate was given by Medical Research Ethics Committee (MREC) Utrecht. The implementation of this approach during the crisis situation of the first wave of the COVID-19 pandemic was according to the Dutch Medical Treatment Contracts Act (WGBO), article 7:458 BW and article 24 and 28 as stated in the General Data Protection Regulation (GDPR) implementing law. The procedures and methods were carried out in accordance with relevant guidelines and regulations.

### Patients

**Fig. 1:**
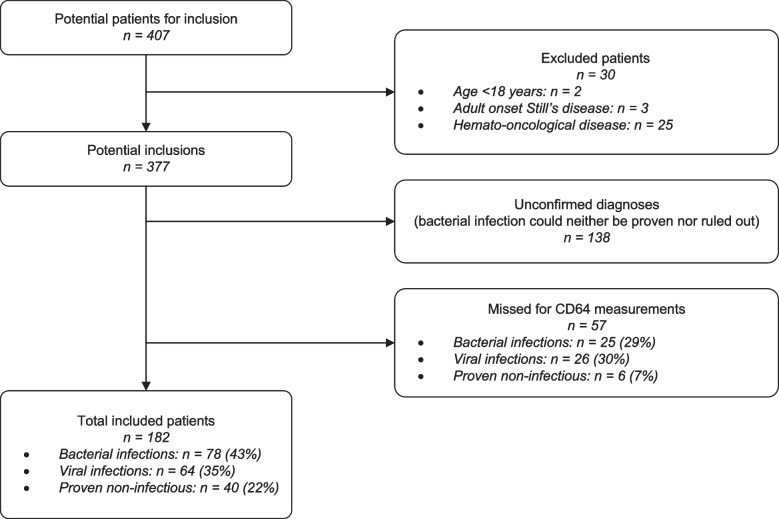
Flowchart of patient inclusion and exclusion

All patients who presented at the ED between March 19th, 2020, and May 17th, 2020, with a suspicion of COVID-19) were enrolled in this study. COVID-19 suspicion during this first phase of the COVID-19 pandemic encompassed almost every patient that came in with any signs that could be due to an infection. This diverse group of potentially infectious patients all underwent a standard diagnostic workup at the ED, including drawing of blood and testing for COVID-19. We excluded patients younger than 18 years old and patients with hematological-oncological diseases (Fig. 1). Patients with adult-onset Still’s disease (adult-onset systemic juvenile idiopathic arthritis) were excluded, as Still’s disease has been reported to affect nCD64 expression [17].

We retrospectively divided the included patients into three mutually exclusive groups based on their final diagnosis as determined by the treating clinical team: ‘bacterial infection’, ‘viral infection’ or ‘non-infectious disease’. Diagnostic work-up for all the patients was initiated by their ED physician who was unaware of the patient’s test results for nCD64 at the time. A bacterial infection was defined as either a positive bacterial test or sufficient radiological evidence, in combination with related clinical symptoms. A positive bacterial test was defined as a positive bacterial culture (blood, sputum, urine, or wound), a positive feces PCR for pathogen (Salmonella spp., Shigella spp., enteroinvasive *E. coli*, *Yersinia Enterocolitica*, Campylobacter, *Plesiomonas Shigelloides*, shiga toxin-producing *E. coli* or enterohemorrhagic *E. coli*) or a positive urinary antigen test (BinaxNow™, Massachusetts, USA), taken during presentation at the ED, or within 2 days of presentation at the ED. Details on how bacterial culture procedures were performed can be found in Additional file 1. The results of these cultures were combined with matching symptoms corresponding to an infection of the specific culture site. An infection was also considered as a bacterial infection if radiological findings combined with the patient’s symptoms made the diagnosis of bacterial infection highly likely, but positive cultures could not be obtained or were deemed inadequate (e.g., a non-drainable abscess on a CT scan).

A viral infection was defined as a positive viral PCR test, taken at the emergency department or within 2 days of admission in combination with matching symptoms. All patients in this study were tested at least once for COVID-19 immediately after presenting at the ED using a SARS-CoV2 specific PCR test from the respiratory tract. A triple-target PCR for the E gene, N gene and the RdRp gene was used. Some patients were also tested for other respiratory viruses at the discretion of their attending physician using a broad range PCR, testing for influenza, RSV, coronaviruses other than SARS-CoV2, and parainfluenza virus (GenMark ePlex® RP2, Roche, Switzerland). Similarly, some patients presenting with complaints of diarrhea, were tested for norovirus or enterovirus using feces PCR. All bacterial and viral cultures and PCR tests were analyzed using the normal, standard-of-care hospital procedures.

Patients who met the criteria for both a bacterial and viral infection (bacterial superinfection) were categorized as a bacterial infection, since we aimed to primarily identify bacterial infections (with or without an extra viral infection) using nCD64.

The non-infectious disease group contained patients with a wide range of non-infectious diagnoses. These patients had symptoms which could definitively be explained by a non-infectious diagnosis (i.e., myocardial infarction, cholelithiasis). Additionally, these patients had to be free of any evidence of a concomitant infection in the days after presentation.

A definitive diagnosis could not be made for a large proportion of patients presented at the ED. This group consisted of patients with an infection of uncertain origin (i.e., no definitive differentiation between viral or bacterial) and patients with a likely non-infectious diagnosis, but an infection could not be completely ruled out. Due to this diagnostic uncertainty, these patients were registered separately and their nCD64 values are described in the results section, but not included in the main analysis.

### Clinical data collection

We collected the following baseline characteristics from the electronic patient files: sex, age, admission to the hospital ward, length of hospital admission in days, potential immunocompromised state, use of antibiotics prior to ED visit and mortality. Furthermore, laboratory values CRP (mg/L) and WBC counts (10^9^ cells/L) were obtained from the patient records.

### nCD64 flow cytometric analysis

Blood was taken from the patients for neutrophil analysis (including nCD64 expression) within 2 h after presentation at the ED. One 4 mL Vacuette® sodium heparin blood tube (Greiner Bio-One, Kremsmünster, Austria) was drawn as part of the standard of care work-up for COVID-19 suspected patients. This tube was directly put into the flow cytometer by ED staff. The flow cytometric method used to analyze nCD64 is similar to the method used to study neutrophil phenotype markers, as validated and described in full extend elsewhere [5]. For this analysis we used the AQUIOS CL® load-and-go automated flow cytometer (Beckman Coulter Life Sciences, FL, USA). This is a completely automated Point-of-Care flow cytometer which can be operated by ED staff without knowledge of flow cytometry. The machine accepted standard blood tubes, which were placed into the machine by the ED staff. The machine then performs the work up, staining and measurement of the blood on a fully automated basis within 15–30 minutes. First, 43 μL of blood was pipetted into a 96-wells plate by the machine, after which the blood was stained with 18 μL of monoclonal antibody mix. This antibody mix included antibodies from Beckman Coulter (Florida, USA): CD64-PC7 clone 22, CD16-FITC (clone 3G8), CD11b-PE (clone Bear1), CD62L-ECD (clone DREG56), CD10-PC5 (clone ALB1) [16]. After fifteen minutes of incubation, 335 μl of lysing reagent A was added to the well: a cyanide-free lytic reagent that lysed red blood cells. 100 μl of lysing reagent B was subsequently added to slow the reaction caused by reagent A and preserve the white blood cells for measurement in the flow cell. The sample was then aspirated for flow cytometric analysis. The entire process including analysis took less than 25 minutes per sample.

Neutrophils were gated using preset gates based on their side- and forward scatter, using the software on the AQUIOS CL® flow cytometer. However, one researcher checked and revised, when necessary, these gates every morning for the samples from the previous day. The expression of nCD64 was reported in the arbitrary unit (AU) of Median Fluorescence Intensity (MFI).

### Statistical analysis

All statistical analyses were conducted in R (Version 3.6.3, R Core team, 2020), and figures were produced using GraphPad Prism version 8 (GraphPad software, Inc., San Diego, CA, USA). Statistical significance was accepted at *p* ≤ 0.05. All tests were performed two-sided. Correlations were calculated using Spearman’s correlation test. Each individual marker was compared between the three categories (bacterial, viral and non-infectious) using the Kruskal-Wallis test. If this test indicated significant differences, post hoc tests were performed using Dunn’s test with a Holm-Bonferroni *p*-value adjustment for multiple comparisons. ROC curves were plotted for all three biomarkers, comparing patients with a confirmed bacterial infection to a control group which consisted of both patients with a confirmed viral infection and patients with a non-infectious inflammatory diagnosis. Optimal cut-offs were calculated using the Youden’s index [18]. Because the optimal cut-off for nCD64 had a low clinical applicability, a second cut-off was calculated which maximized the positive predictive value to one, as it was hypothesized that nCD64 includes bacterial infections, but not necessarily excludes them. Lastly, patients with bacterial infections were further analyzed in order to see whether the culture type, disease severity (estimated using the Quick Sofa (qSOFA) Score [19]) or use of antibiotics had an effect on nCD64 AU MFI values.

### Diagnostic algorithm

We developed a prototype for a diagnostic algorithm to identifying patients with bacterial infections. We incorporated a nCD64 value in this algorithm which corresponded with a PPV of one, indicating a definite bacterial infection. As this high PPV came with a loss of sensitivity, the optimal cut-offs of CRP and WBC counts were then used to identify the remaining patients with bacterial infections.

## Results

### Patient inclusion and baseline characteristics

Four hundred seven COVID-19 suspected patients visited the ED of the University Medical Center Utrecht as a tertiary referral center between March 19th and May 17th, 2020. They were all tested for (and thus suspected of) having COVID-19. Thirty patients were excluded because they met the exclusion criteria (age < 16, *n* = 2; adult-onset Still’s disease, *n* = 3; hemato-oncological disease, *n* = 25). Another 138 patients were excluded because no definitive diagnosis could be proven nor ruled out. Due to several reasons, 57 cases were missed for nCD64 analysis. Examples of these reasons were: no blood samples taken from the patient, blood sample tube was put incorrectly in the flow-cytometer, ineffective erythrocyte lysis or incorrect analysis due to a malfunction of the flow cytometer. In total, 182 patients with a definitive diagnosis and adequate nCD64 measurement were included in this analysis. 78 patients (43%) had a proven bacterial infection, 64 (35%) had a proven viral infection (58 of which had COVID-19) and 40 (22%) had a non-infectious disease. This distribution did not differ significantly from the 57 patients that were missed (Fig. 1). The three groups did not differ in baseline characteristics or mortality rate (Table 1). Patients with either a bacterial or viral infection were more often admitted to the hospital and were admitted longer than patients with a non-infectious disease.

**Table 1:** Baseline characteristics of included patients

	Bacterial infections	Viral infections	Non-infectious disease:	Total (*p*)
*N (%)*	78 (43%)	64 (35%)	40 (22%)	182
Sex (Male/Female)	43/35	33/31	19/21	96/87 (*p =* 0.73)
Median age in years (min – max)	66 (18–95)	63 (29–91)	61 (23–85)	63 (18–95) (*p =* 0.14)
*N* hospital admissions (%)	65 (83.3%)	52 (82.5%)	21 (51.2%)	138 (*p* < 0.001)
Median length of admission in days (min – max)	5 (0–61)	6 (0–39)	1 (0–36)	4 (0–61) (*p* < 0.001)
*N* Immunocompromised patients (%)	29 (37.2%)	17 (26.6%)	17 (42.5%)	63 (34.6%) (*p* = 0.21)
*N* patients already using antibiotics (%)	16 (20.5%)	8 (12.7%)	2 (4.9%)	26 (14.3%) (*p* = 0.06*)*
*N* deaths	8 (10.3%)	10 (15.6%)	3 (7.5%)	21 (11.5%) (*p* = 0.40)

Group definitions. Bacterial infections: Patients with symptoms suggestive of infection, combined with either a positive bacterial culture or radiological evidence of bacterial infection. Viral infections: Patients with symptoms suggestive of infection, combined with a positive viral PCR test. Non-infectious disease: Patients with symptoms matching a non-infectious diagnosis, without evidence of any infection according to their physician. Nb. Patients with both a bacterial infection and a second diagnosis are grouped under bacterial infections

### Biomarkers in bacterial infection

**Fig. 2:**
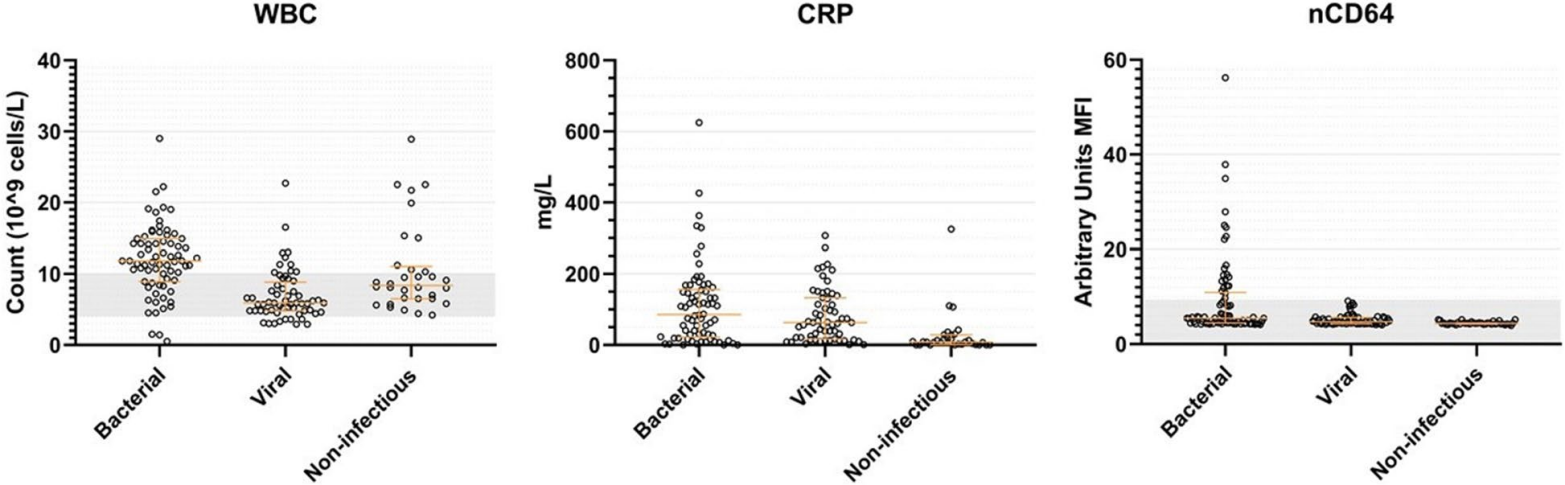
Distribution of WBC counts, CRP and nCD64 in AU MFI per diagnosis. Single data points with median and interquartile ranges are shown. For WBC counts the grey area represents physiological values. For nCD64 the grey area represents the values below 9.4 AU MFI

WBC counts differed among the three categories (χ^2^: 40.7, df = 2, *p <* 0.0001). Median counts were 11.8·10^9^ cells/L for the bacterial group, 5.9·10^9^ cells/L in the viral group and 8.4·10^9^ cells/L for the non-infectious inflammatory disease. A post hoc test indicated that all groups differed significantly from each other (*p* < 0.05; Fig. 2).

CRP differed significantly among the groups (χ^2^: 29.9, df = 2, *p <* 0.0001), with a higher CRP value in both the bacterial group (median 86 mg/L) and the viral group (median 63 mg/L), compared to the non-infectious group (median 8 mg/L). Post hoc testing showed no differences between the bacterial and the viral group (*p* = 0.41, Fig. 2).

The median nCD64 MFI for the bacterial, viral and non-infectious groups were 5.5, 4.7 and 4.4, respectively (χ^2^: 33.9, df = 2, *p <* 0.0001). The median nCD64 MFI was higher in the bacterial group compared to viral disease group (*p* = 0.004) and non-infectious disease group (*p* < 0.0001). nCD64 was also higher for viral infections versus non-infectious diseases (*p* = 0.003, Fig. 2). The nCD64 MFI values had a wider range in the bacterial group (4.14–56.23) compared to the other groups (4.1–9.14 and 3.92–5.19, respectively).

WBC counts and CRP were found to have a weak positive correlation (*r*_*s*_ = 0.19, *p =* 0.02), and CRP was found to have a moderate positive correlation with nCD64 (*r*_*s*_ = 0.56, *p* < 0.001). No correlation was found for WBC counts and nCD64 (Table 2).

**Table 2:** Correlation of biomarkers using Spearman’s rank correlation coefficient

Biomarker	WBC	CRP	nCD64
WBC	1	X	X
CRP	0.19 (*p* = 0.02)	1	X
nCD64	0.12 (*p* = 0.14)	0.56 (*p* < 0.001)	1

### nCD64 can reliably rule in, but not rule out bacterial infections

**Fig. 3:**
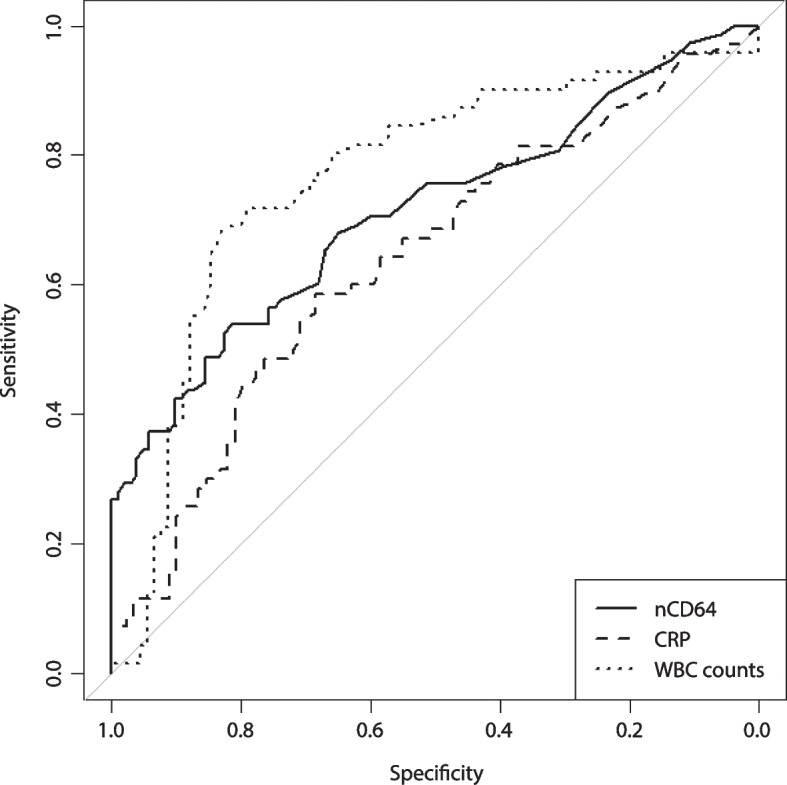
ROC-curves of the three biomarkers: nCD64, CRP and WBC counts

We plotted ROC curves for nCD64, CRP and WBC, comparing patients with bacterial infections to a control group consisting of the viral and non-infectious groups. No significant differences were found in the AUC of nCD64 (AUC: 0.71 [*95% CI*: 0.64–0.79]) compared to the AUCs of WBC counts (AUC: 0.77 [*95% CI*: 0.69–0.84]) and CRP (AUC: 0.64 [*95% CI*: 0.55–0.73]) (Fig. 3). The optimal cut-offs for each parameter were calculated using the Youden index and these are shown in Table 3. In addition to the Youden index, we also investigated at which MFI nCD64 had a positive predictive value of one, as nCD64 is hypothesized to positively identify patients with bacterial infections, but cannot necessarily exclude these infections. Using a MFI cut-off point of 9.4 AU (an increase of 102% compared to the nCD64 of patients without an infection), a specificity of 1.00, sensitivity of 0.27, PPV of 1.00 and NPV of 0.64 were calculated (Table 3).

**Table 3:** Clinical performance of biomarkers in diagnosing bacterial infections

Biomarker	Method	Threshold (reference range)	Sensitivity (95%-CI)	Specificity (95%-CI)	Positive Predictive Value (95%-CI)	Negative Predictive Value (95%-CI)
nCD64 (AU MFI)	Youden’s index	5.4	0.54	0.82	0.69	0.70
nCD64 (AU MFI)	Maximized specificity/PPV	9.4	0.27	1.00	1.00	0.64
CRP (mg/L)	Youden’s index	68.5 (0–10)	0.59	0.69	0.59	0.68
CRP (mg/L)	Maximized specificity/PPV	327.0 (0–10)	0.07	1.00	1.00	0.58
WBC (·10^9^ cells/L)	Youden’s index	10.4 (4–10)	0.69	0.82	0.75	0.77
WBC (·10^9^ cells/L)	Maximized specificity/PPV	29.0 (4–10)	0.01	1.00	1.00	0.57

For each biomarker, two optimal cut-off points were calculated: 1) using the Youden index; 2) By maximizing specificity and the PPV. For CRP and WBC counts, the reference range used by our hospital laboratory are shown

Patients with bacterial infections were further analyzed to explain the wide range of nCD64 results. No differences in nCD64 MFI were found between three different disease severity groups based on the qSOFA score (χ^2^: 3.6, df = 2, *p =* 0.16) (Additional file 2A). The study lacked the statistical power to test for differences between bacterial species. We found a trend towards lower nCD64 values for patients already on antibiotics, which did not reach statistical significance (median nCD64: 5.73 vs. 4.83, χ^2^: 2.7, df = 1, *p =* 0.10) (Additional file 2B).

As nCD64 at a cut-off of 9.4 AU MFI had a high PPV and specificity, but a low sensitivity, we retrospectively created a prototype for a diagnostic algorithm in order to identify bacterial infections, even in patients with low nCD64 values (Additional file 3). Only patients for which nCD64, CRP and WBC were all available, were included (*n* = 159). Although this algorithm has a high positive predictive value for diagnosing bacterial infections (88%), the low sensitivity (42.9%) means it likely has little clinical applicability in a broad population yet.

### nCD64 in patients with an unconfirmed disease

**Fig. 4:**
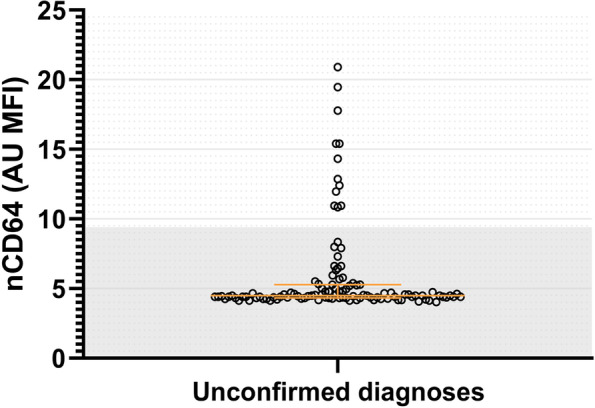
nCD64 values in patients with unconfirmed diagnoses. Note that several patients with an unconfirmed diagnosis have an nCD64 > 9.4

nCD64 was also determined in patients without a definitive diagnosis, despite the fact that these patients were not included in the main analyses. The median MFI of nCD64 in this group was 4.5 AU. Although most of these patients (91.3%) had nCD64 MFI values below 9.4 AU, 12 patients (8.7%) had nCD64 MFI values higher than 9.4 AU (Fig. 4).

## Discussion

This study was carried out in patients at the ED of a large academic hospital who presented with signs of an infection. We found an increased expression of nCD64 in patients with bacterial infections when compared to viral infections and non-infectious inflammatory diseases. A relatively high MFI cut-off value for nCD64 (9.4 AU) on this specific automated flow cytometer can be used as a rule in test for bacterial infections, as both the specificity and PPV were 1.0. Interestingly, the nCD64 MFI was higher than 9.4 AU in 8.7% of the patients who got excluded because of their unconfirmed diagnoses. This indicates that in this group bacterial diagnoses might have been missed and that nCD64 could have contributed to detection of these cases. However, with a sensitivity of 0.27 and NPV of 0.64, this threshold of nCD64 MFI is not a sufficient rule out test.

In the present study we found a similar AUC for nCD64 compared to WBC and CRP (AUCs: 0.64–0.77). An optimal MFI cut-off point (5.4 AU) for nCD64 was calculated using the Youden index [18]. As this corresponded with a low sensitivity and a moderate specificity, this cut-off was not sufficient to include or exclude bacterial infections. nCD64 was hypothesized to include, but not exclude, bacterial infections. Therefore, a new cut-off point was identified which maximized the PPV to a value of 1, but which decreased the sensitivity to 0.27. This only allows for a subset of bacterial infections to be diagnosed, but this diagnosis can be made with great certainty. On the other hand, maximizing the PPV to 1 for CRP and WBC counts would lead to very high cut-off values, which would be accompanied by such low sensitivity that it would have no clinical relevance (Table 3). The low sensitivity of nCD64 in this study is in contrast with the results found in recent meta-analyses [7, 11, 13, 14]. This might be due to the differences in patient groups, as most patients in the meta-analyses were admitted to the ICU with a severe critical illness such as sepsis. A higher diagnostic yield is likely when nCD64 is used in a critically ill ICU population suspected of bacterial infection. In contrast, we analyzed nCD64 in a more heterogenic ED population with a wide variety in disease severity and differences in time in the course of the disease.

The diverse study population might explain the wide range of nCD64 values. As reported, there might be a difference in nCD64 values related to disease severity, which most likely differs compared to studies conducted in an ICU setting. Another explanation for the variation in nCD64 values could be the range in the delay in presentation on the ED after onset of symptoms [15]. It is shown that nCD64 upregulation and downregulation after onset of disease is dependent on time [9, 15]. Longitudinal measurements might bypass this time dependency effect and improve the diagnostic yield of nCD64 for bacterial infections [15]. It is unknown if infections with different pathogens cause different responses in terms of nCD64 upregulation. Previous ex vivo research on this subject shows mixed results. Akhmaltdinova et al. [20] found a similar nCD64 among gram positive and negative infections, while Herra et al. [21] found that infections caused by gram negative bacilli induced a higher nCD64 expression than other bacterial infections. In the present study, a subgroup analysis into nCD64 values amongst different bacteria lacked the power for valid statistical analysis. Some patients in our study population already received antibiotics that were prescribed by the general practitioner. Previous meta-analyses demonstrated lower sensitivity values in studies that did not exclude use of antibiotics prior to nCD64 measurement [7]. Indeed, patients in the bacterial infection group in our population who were on antibiotics showed a trend towards lower nCD64 values (Additional file 2B). Despite the fact that our results were not significantly different, it might still be that antibiotic use has a negative effect on the nCD64 test results, also when taking into account previous literature [7]. Nevertheless, despite this wide range of patients and symptoms, nCD64 could still rule-in bacterial infections, which adds to the generalizability of the use of nCD64 as a diagnostic test.

A combination of multiple biomarkers could lead to an improved diagnostic value compared to a single marker [22, 23]. We proposed a diagnostic algorithm, which added WBC counts and CRP into the decision making, in order to identify bacterial infections in patients with a nCD64 AU MFI lower than 9.4 (Additional file 3). This algorithm was able to rule in more bacterial infections than nCD64 alone, but could still not sufficiently rule these out. Our diagnostic algorithm is therefore suboptimal in this broad population and serves as an example of what future diagnostic algorithms could look like, instead of having a high clinical applicability. In order to produce an applicable algorithm, there is a need for biomarkers that complement the diagnostic accuracy of nCD64. Future biomarkers should either identify bacterial infections in patients with a low nCD64, or make other diagnoses more likely. A potential biomarker is CD169, which in normal circumstances is expressed on monocytes at low levels. CD169 becomes strongly overexpressed on monocytes in response to a viral infection, such as SARS-CoV-2, HIV, or the common flu [24–26]. Velly et al. [27] found that the combination of HLA-DR on monocytes, MerTK on neutrophils and plasma metalloproteinase-9 could accurately distinguish bacterial infections from viral infections and other inflammatory disease. There are multiple potential candidate biomarkers for a more extensive diagnostic algorithm in combination with nCD64, but additional studies are necessary to explore this concept in the ED setting.

This study has its limitations, of which the heterogeneity of the studied population is the most important. This positively impacts generalizability, but hampers the diagnostic yield of the biomarker. The large heterogeneity resulted in a relatively small number of patients per category and the exclusion of a large number of patients without definitive diagnoses. However, this analysis still showed nCD64 as a biomarker with added value to diagnose bacterial infections. Also adding to the heterogeneity is the complicated population with multiple comorbidities, typically seen in tertiary referral hospitals. This has led to a relatively large number of patients that did not receive a definitive diagnosis in our study population. Future research should also include patients from regional hospitals and general practices in order to bypass this problem. Another limitation of this study is the overrepresentation of COVID-19 cases. Consequently, other viral infections are underrepresented, which could have caused sampling bias. However, previous research showed that nCD64 was rarely upregulated both in a cohort of patients with COVID-19 and a cohort of patients with other viral infections [23]. Although nCD64 can be upregulated in patients with viral infections, this effect is generally lower than in bacterial infections [10, 28]. Interestingly, Velly et al. [27] found higher nCD64 values in patients with viral infections than in patients with bacterial infections. This result is not replicated in our study, nor in other research to our knowledge. Future research is needed to clearly identify any differences in nCD64 expression between different viral infections.

## Conclusion

Neutrophil CD64 measured with fully automated Point-of-Care flow cytometry in the ED, has a potential as a rule in test to diagnose bacterial infections, but has an insufficient negative predictive value. The combination of nCD64 with other (new) biomarkers is needed to construct algorithms that will adequately aid ED clinicians to diagnose bacterial infections.

## Supplementary Information

**Additional file 1. **Details of the bacterial culture procedures.

**Additional file 2. **Exploratory analysis amongst patients with bacterial infections regarding the QSOFA score and nCD64 expression (Additional file 2A) and antibiotic use and nCD64 expression (Additional file 2B).

**Additional file 3. **Prototype of a diagnostic algorithm constructed using the data from this study.

## Data Availability

The datasets used and/or analysed during the current study are available from the corresponding author on reasonable request.

## Abbreviations

AU: Arbitrary Units
AUC: Area Under the Curve
CRP: C-Reactive Protein
ED: Emergency Department
FcɣR1: Fc-gamma-receptor-1
IgG: Immunoglobulin-G
G-CSF: Granulocyte Colony Stimulating Factor
ICU: Intensive Care Unit
IQR: Interquartile range
MFI: Median Fluorescent Intensity
nCD64: Neutrophil CD64
NPV: Negative Predictive Value
PCR: Polymerase Chain Reaction
POC: Point-of-Care
PPV: Positive Predictive Value
ROC: Receiver Operating Characteristic
WBC: White Blood Cell
