# Supplementary material for: Point-of-care neutrophil CD64 as a rule in diagnostic test for bacterial infections in the emergency department
Source: BMC Emerg Med. 2023 Mar 14;23:28. doi: 10.1186/s12873-023-00800-2 (PMC10010956; doi:10.1186/s12873-023-00800-2)
Supplement: Supplementary file 1 — Additional file 1. Details of the bacterial culture procedures. [file 12873_2023_800_MOESM1_ESM.docx]

**Additional File 1.** Details of the bacterial culture procedures.

Blood cultures were drawn using BacTec™ (BD Diagnostics, Sparks, MD) blood culture bottles in cases where patients had a fever above 38.5 degrees Celsius or when a bacteremia was suspected. For each patient, two sets of blood cultures were drawn from two separate venipuncture sites. Each set consisted of an aerobic bottle and an anaerobic bottle. The aerobic bottle was drawn before the anaerobic bottle. If growth was detected, the culture was deemed positive and a Gram stain was performed. Then the blood was subcultured to further identify the pathogen and test for antibiotic susceptibility. Provisional results were generally available within one or two days, while definitive results were available after five to eight days.

Sputum cultures were taken if the patient had a sputum-producing cough. Sputum was then collected in a sterile container, a Gram stain was performed and the sample was cultured for pathogenic micro-organisms using the standard procedures. Definitive results were generally available after two to five days.

Urine cultures were taken either using a midstream urine sample or from a catheter and were collected in a sterile container. Like the sputum culture, a gram stain was performed and the sample was cultured using standard hospital procedures. The results of the urine culture were generally available within one to four days.

Wound cultures were taken using samples from potentially infected wounds, either collected using a liquid culture swab (Sigma Transwab®, MWE, Corsham, UK) or by collecting pus in a sterile container.

Blood cultures were incubated immediately after collection, while sputum, urine and wound cultures were incubated only during office-hours. Samples collected after office hours were kept in a fridge until they were incubated the following day.

Pathogen identification was done as previously described by Hellebrekers *et al.* 2019. (1)

**References**

1. Hellebrekers P, Rentenaar RJ, McNally MA, Hietbrink F, Houwert RM, Leenen LPH, et al. Getting it right first time: The importance of a structured tissue sampling protocol for diagnosing fracture-related infections. Injury. 2019;50(10):1649–55.
