# Supplementary material for: Point-of-care neutrophil CD64 as a rule in diagnostic test for bacterial infections in the emergency department
Source: BMC Emerg Med. 2023 Mar 14;23:28. doi: 10.1186/s12873-023-00800-2 (PMC10010956; doi:10.1186/s12873-023-00800-2)
Supplement: Supplementary file 2 — Additional file 2. Exploratory analysis amongst patients with bacterial infections regarding the QSOFA score and nCD64 expression (Additional file 2A) and antibiotic use and nCD64 expression (Additional file 2B). [file 12873_2023_800_MOESM2_ESM.docx]

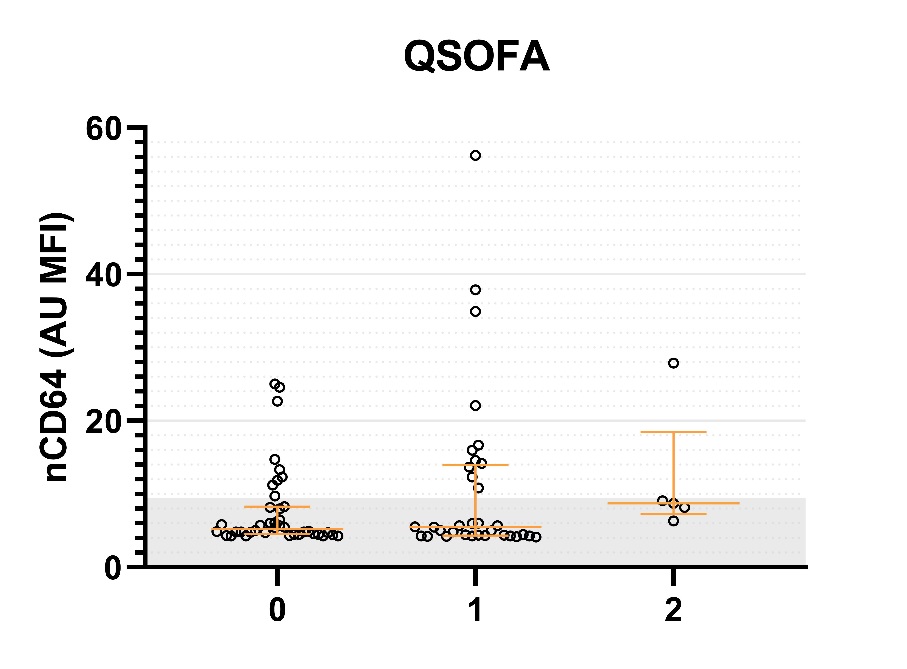


**Additional File 2A**. Exploratory analysis amongst patients with bacterial infections. Additional File 2A shows the nCD64 values compared to disease severity (measured using the qSOFA Score for patients at risk of sepsis/poor outcome, ranging from 0 to 3 (1)). No significant differences were found in nCD64 scores between patients with different qSOFA scores (*p*=0.16).

**References**

1. Seymour CW, Liu VX, Iwashyna TJ, Brunkhorst FM, Rea TD, Scherag A, et al. Assessment of clinical criteria for sepsis for the third international consensus definitions for sepsis and septic shock (sepsis-3). JAMA - Journal of the American Medical Association. 2016;315(8):762–74.


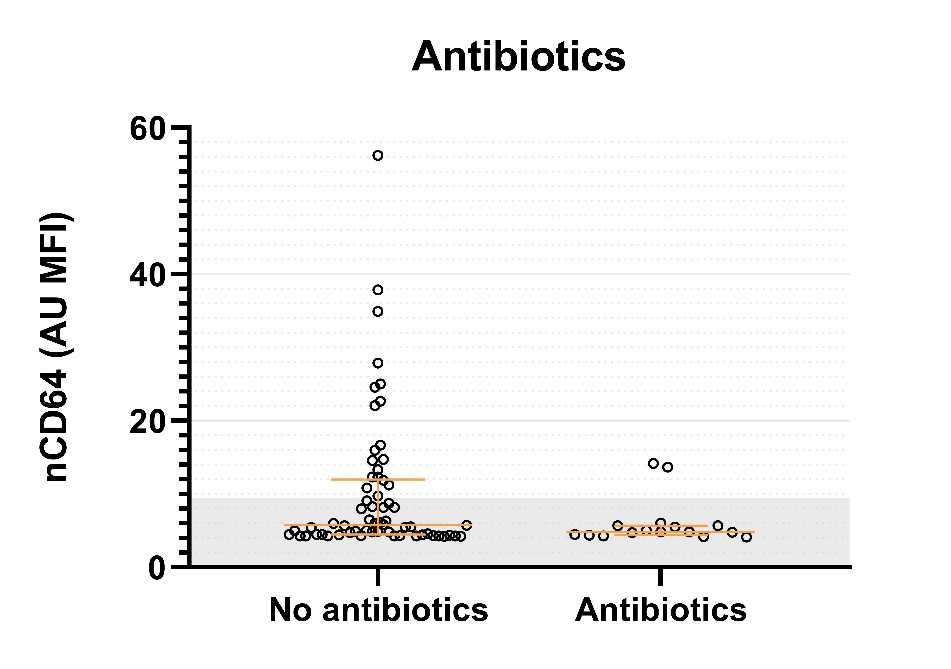


**Additional File 2B.** Exploratory analysis amongst patients with bacterial infections. Additional File 2B shows the nCD64 values of patients who had already started antibiotic treatment prior to presentation at the ED. No significant difference was found (*p=*0.10, two-sided).
