# Supplementary material for: Point-of-care neutrophil CD64 as a rule in diagnostic test for bacterial infections in the emergency department
Source: BMC Emerg Med. 2023 Mar 14;23:28. doi: 10.1186/s12873-023-00800-2 (PMC10010956; doi:10.1186/s12873-023-00800-2)
Supplement: Supplementary file 3 — Additional file 3. Prototype of a diagnostic algorithm constructed using the data from this study. [file 12873_2023_800_MOESM3_ESM.docx]

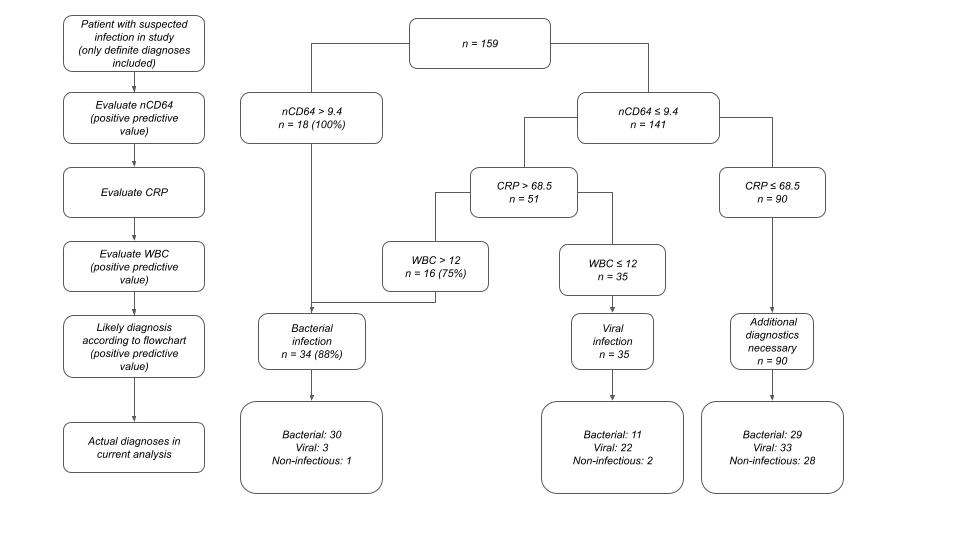


**Additional File 3.** Prototype of a diagnostic algorithm constructed using the data from this study. The goal of this algorithm is to serve as an example of what future prediction models could look like and what type of biomarkers are still needed to optimize diagnostics. For all patients, nCD64, CRP and WBC were determined upon presentation in the ED. This algorithm first identifies patients with a nCD64 >9.4 AU MFI, as these were all patients with a bacterial infection (n=18). In order to identify bacterial infections in patients with nCD64 <9.4 AU MFI, CRP (>68.5mg/L) was used to identify those suspected of an infection. Among these patients, WBC counts further differentiated between bacterial (>10.4*10⁹cells/L) and viral (<10.4*10^9^ cells/L) infections. In 141 patients with an nCD64 ≤9.4, 51 patients had a CRP >68.5, of which 16 had a WBC count >12, indicating a bacterial infection. In total, 34 patients were identified by this algorithm as a bacterial infection, of which 30 (88.2%) had a confirmed bacterial infection. However, of the 70 included bacterial infections, only 30 (42.9%) were correctly identified. Of the 35 patients identified as a viral infection by this algorithm, 22 had a confirmed viral infection (62.9%). The algorithm was unable to aid in the diagnosis in 90 cases (56.6%). For these cases, further diagnostics would be necessary.

This algorithm suggests that in patients with an nCD64 > 9.4, bacterial infection is certain, with a PPV of 100%, even when CRP an WBC counts are within normal limits. In patients with an nCD64 <9.4, a bacterial infection is still likely if CRP > 68.5 and WBC >12 (PPV: 75%). In these patients, antibiotic therapy is likely to be warranted. This algorithm would have diagnosed 34 bacterial infections, of which 30 patients had an actual bacterial infection (PPV = 88%). Although 40 cases of bacterial infections were missed (57%), meaning there is little clinical applicability and additional biomarkers are needed to make a bacterial infection diagnosis more reliable.
